# Intermittent meromixis controls the trophic state of warming deep lakes

**DOI:** 10.1038/s41598-020-69721-5

**Published:** 2020-07-31

**Authors:** Maximilian P. Lau, Giulia Valerio, Marco Pilotti, Michael Hupfer

**Affiliations:** 1 Department of Chemical Analysis and Biogeochemistry, Leibniz Institute of Freshwater Ecology and Inland Fisheries (IGB), Müggelseedamm 301, 12587 Berlin, Germany; 2 DICATAM, Università Degli Studi di Brescia, Via Branze 43, 25123 Brescia, Italy; 3 Département des sciences biologiques, Université du Québèc à Montréal (UQAM), 141, avenue du Président-Kennedy, Montréal, QC H2X 1Y4 Canada

**Keywords:** Limnology, Biogeochemistry, Limnology, Element cycles

## Abstract

Vertical mixing modulates nutrient dynamics in lakes. However, surface warming reduces the range of vertical mixing and the probability of full circulation events. Important consequences of reduced vertical mixing include the sequestration of phosphorus (P) within a stagnant zone and the promotion of oligotrophication. Nevertheless, warming-induced shifts from full to partial mixing (meromixis) are not permanent and are partially reversible during exceptionally cold or windy winters. In this study, we investigated how intermittent meromixis affects lake P budgets. We examined the P cycle of a perialpine lake with variable mixing depths by pairing sedimentation and release flux measurements with sedimentary archives. We found that the amount of dissolved P surpassed that of the potentially mobile P in the sediments by a 13:1 ratio. At least 55% of the settled P was rapidly released to bottom waters isolated from flushing, illustrating the general biogeochemical mechanism that promotes deep-water P storage when lakes undergo warming. This storage process is abruptly inverted when meromixis suddenly retreats, deeper mixing introduces P pulses to the surface waters, thereby promoting phytoplankton proliferation. Our estimates showed that lakes containing up to 40% of the global freshwater volume could shift towards intermittent meromixis if the atmospheric warming trend continues. Thus, these lakes might accumulate 0–83% of their P load in irregularly circulating waters and are prone to large P pulses.

## Introduction

The transport of excess nutrients to surface waters is symptomatic of anthropogenic changes occurring in the biosphere^1,2^. Eutrophic lakes overproduce phytoplankton and their settling dead biomass depletes hypolimnetic oxygen^3^. Bottom water anoxia limits the habitats of all aerobic biota and modulates the biogeochemical processes determining the fate of nutrients, trace metals, and greenhouse gases^4–6^. Phosphorus (P) is a limiting nutrient in most freshwater systems^7^ and particular attention has thus been directed towards the management of urban and agricultural P export. However, the improvements in oxygen supply realised by reducing P loading might be offset by another anthropogenic stressor: increases in lakewater temperature^8–10^.

The global mean surface temperature has increased by 0.78 ± 0.06 °C over the last century^11^. There is clear evidence that lake surface temperatures have also followed a warming trend^12^. It has been forecasted that lake surface temperatures will increase by ≥ 70% of the anticipated increase in air temperature^13^. Warm lakes are at an elevated and growing risk of bottom-water anoxia because of enhanced phytoplankton growth and organic matter mineralisation^14,15^, increasing thermal stability, and prolongation of the stratification period^16–19^. Warming of deep dimictic or monomictic lakes may cause them to shift to oligomictic regimes wherein the lakes no longer undergo full mixing each year^6,20,21^. During stagnation, the ongoing release of mineralisation products stabilises density stratification and eventually shifts these lakes towards a ‘biogenic’ meromictic regime wherein the deepest layer of the water, the monimolimnion, presents with perennial stagnation^22–24^.

It is expected that changes in mixing regime profoundly alter internal nutrient cycling. Protracted stagnation may lower the redox-sensitive P binding capacity^25^, extend the time available for mineralisation in stagnant bottom waters, thereby increasing the internal P supply to the euphotic zone during holomixis^26^. Opposed to that, partial mixing in a meromictic lake furnishes only parts of sediment-released P to the euphotic zone^27^ and some P remains trapped in the monimolimnion^28^. Warming-driven transitions towards meromixis might counterintuitively modulate the fate of P by withdrawing some of the settled P from the seasonal cycle. Several studies reported that phytoplankton communities adapt to nutrient deficiencies in response to changes in deep mixing patterns^29–31^. These nonlinear reactions to warming that promote nutrient sequestration during changes in mixing patterns were previously predicted^32–34^ but the ecological consequences involved have seldom been explored^27,35^.

Here, we investigated the effects of a reduction in deep circulation on nutrient cycles in deep lakes. Meromixis occurs when the density stratification of a lake impedes mixing, which is itself driven by atmospheric forcing such as wind and cooling. We propose that progressive warming of the water promotes meromixis. However, the current perception of climate-driven nutrient distribution changes in lakes inadequately reflects this effect. The vulnerability of deep temperate-zone lakes to the formation and intensification of meromixis has been previously discussed^21,32,33,36^. Meromixis often occurs in lakes with a relative depth of > 5%^37,38^. Nevertheless, global warming may invalidate this threshold^20^. The prevalence of climate-driven meromixis could increase with decreasing threshold but the effects of the former on lake nutrient balances are unknown.

An aim of the present study was to understand how a shift towards meromixis impacts lake-wide P budgets. The research was conducted on Lake Iseo which is a 256-m perialpine lake in Italy, the mixing depths of which have decreased over time^39,40^. We measured water column and sediment phosphorus pools, related them to their accompanying vertical fluxes (sedimentation and sediment release) during 2016/2017, and set these data within a historic context to identify the internal biogeochemical factors regulating P dynamics. Other objectives of this work included addressing the consequences of climate-driven shifts in mixing regime to lake water quality and globally estimating the general prevalence and magnitude of this phenomenon.

## Results

**Figure 1:**
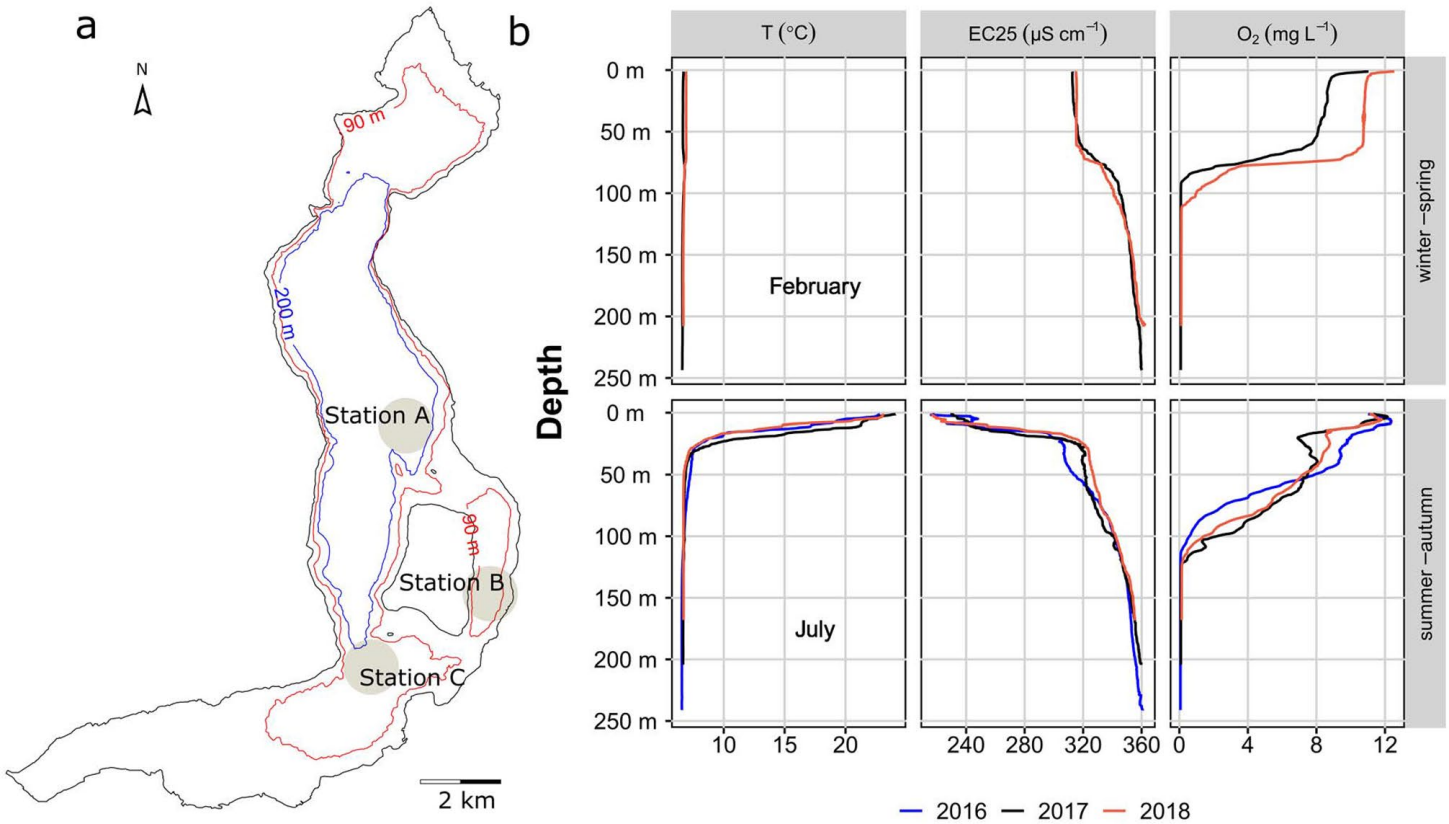
Map and selected characteristics of Lake Iseo. Panel (**a**) depicts the shoreline of Lake Iseo along with two isodepth lines (90 m in red and 200 m in blue). Map was created by use of R software (Version 3.4.3) from published bathymetric data^47,77^. Labeled dots indicate sites sampled throughout the campaigns. Panels (**b**) show temperature (T), electrical conductivity (as 25°C, EC25) and dissolved oxygen (O_2_) distribution in the water column between 2016 and 2018 in the deep basin (station A). Upper and lower row of panels show profiles for the isothermic (February), and summer stratified condition (July), respectively.

Lake Iseo is wide and deep (A = 61 km^2^; z_max_ = 256 m). It is situated in a pre-alpine region of Italy and has a theoretical water retention time of 4.5 year. Its bathymetry is characterised by an extensive deep basin stretching along the central basin north of the island of Monte Isola (Fig. 1a). The sampling locations (A–C) were located in the central sub-basin around Monte Isola. Each one had a different water depth but all were > 90 m.

Temperature, conductivity, and oxygen profiles indicated that the water column was divided into 2–3 compartments the vertical range of which varied annually and seasonally (Fig. 1b). In winter, a uniform temperature and conductivity zone visibly separated the deep monimolimnion characterised by a downward increase in conductivity and anoxia at depths > 100 m. In summer, temperature stratification separated the warmer epilimnion from the cooler hypolimnion. The former comprised a surface isothermal mixed layer and its vertical extent varied with wind velocity and air temperature. However, it never extended below 20 m during summer stratification.

**Figure 2:**
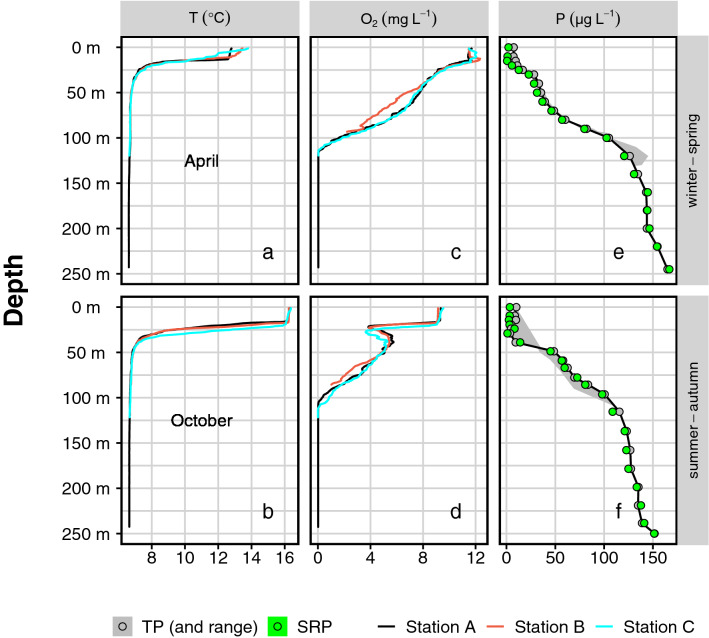
Selected physicochemical properties of Lake Iseo’s water column. Water column profiles of temperature (T, panels **a**/**b**), and dissolved oxygen (O_2_, panels **c**/**d**) were obtained from the three measurement stations A-C (colors). The symbols in panels (**e**/**f**) show concentration of total (TP, grey) and soluble reactive phosphorus (SRP, green) in the water column at Station A. The grey shaded areas show the range of TP across the three measured stations. Upper and lower row of all panels show profiles taken in April and October of 2017, respectively.

The vertical profiles of temperature, oxygen and the total and reactive phosphorus were measured at stations A–C in April and October 2017 (Fig. 2a–f). The soluble reactive P fraction (SRP) was lower than the total P (TP) near the surface. Nevertheless, both were virtually identical in deeper waters. Hence, the latter lacked biomass (phytoplankton and zooplankton) and suspended particles. Increasing proximity to the sediment coincided with a slight increase in TP at all three locations (TP range; Fig. 2e/f). Our estimates for the P dynamics of Lake Iseo were based on a fixed vertical division of the lake into 1-m horizons merged into three discrete compartments. The first was a surface layer in the range of 0–21 m. It represented an euphotic zone where SRP is assimilated during primary production. The second was a middle layer in the range of 21–91 m that usually homogenised with the surface layer in winter. The third was a deep stagnant layer in the range of 91–256 m, called "monimolimnion", the oxygen concentration of which would only slightly increase close to its surface. Therefore, it did not substantially exchange with the surface waters during the sampling period.

Based on our measurements for April–October 2017, we calculated the pelagic TP pools for the three water column compartments and determined the cumulative TP content (Table 1). The steep TP concentration gradient and the concave bathymetry of Lake Iseo disclosed that the most extensive P pool was in the deep compartment (91–256 m; 438–476 t; Table 1) and it far exceeded the P levels in the water layers above it. The TP pool in the surface compartment was very small (~ 2%) compared to that of the deep compartment. However, the top 21 m represented ~ 15% of the total water volume of Lake Iseo (Table 1).

**Table 1:** Selected characteristics and lake-internal phosphorus (P) pools and fluxes at three measurement stations in Lake Iseo (2016–2017).

		Station A	Station B	Station C	Monimolimnion-wide	Lake-wide
**Position**						
Depth at station (represents sub-basin)	M	255	100	130	91–256	0–256
		Central	East Channel	South		
Area (surface)	km^2^	42.2	6.9	11.8		61.1
Area at 91 m (% of Monimolimnion)^a^	km^2^	29.1 (80%)	2.63 (7%)	4.79 (13%)	36.6 (100%)	
Volume	10^6^ m^3^				3,595	7,905
**TP in water column 2017**						
Surface layer, 0–21 m	TP: t P					10.0^b^
	Vol.: 10^6^ m^3^					1,180
Middle layer, 21–91 m	TP: t P					131.4^b^
	Vol.: 10^6^ m^3^					3,176
Monimolimnion, 91–256 m	TP: t P					456.9^b^
	Vol.: 10^6^ m^3^					3,558
**P sedimentation traps**						
P sedimentation flux, 20 m	mg m^−2^ day^−1^		1.89			
P sedimentation flux, 90 m	mg m^−2^ day^−1^		2.01			
**P sediment accumulation**^c^						
Mobile P pool (gradient method, 11 cm)	g m^−2^	1.3	1.0	1.0	1.22	
	t P	37.5	2.6	4.6	44.7	
Mobile P pool (in mobile fractions, 0–5 cm)^d^	g m^−2^	3.1	1.1	1.1	2.68	
	t P	89.9	2.8	5.1	97.9	
Total P pool (11 cm)	g m^−2^	17.7	6.8	7.0	15.5	
	t P	515	18	34	567	
**P sediment release**						
P release (April 2016)^e^	mg m^−2^ day^−1^	2.58	1.73	1.78		
P release (October 2016)^e^	mg m^−2^ day^−1^	1.90	2.80	1.26		
P release (average)	mg m^−2^ day^−1^	2.24	2.26	1.52	2.15	
	t year^−1^	23.8	2.2	2.7	28.7	
**Sediment mineralization**						
F_red_^f^	g O_2_ m^−2^ day^−1^	0.24	0.30	0.20		

^a^Hypsographic curves of sub basins in Supplementary Figure S1, ^b^averages from two sampling time points, ^c^expressed relative to area, cumulative mass profiles for all stations in the Supplementary Figure S3, ^d^calculated according to Hupfer et al.^70^, ^e^from porewater SRP gradients in dialysis samplers, ^f^release of CH_4_, Fe^2+^, Mn^2+^, NH_4_^+^ expressed in oxygen equivalents^15^.

**Figure 3:**
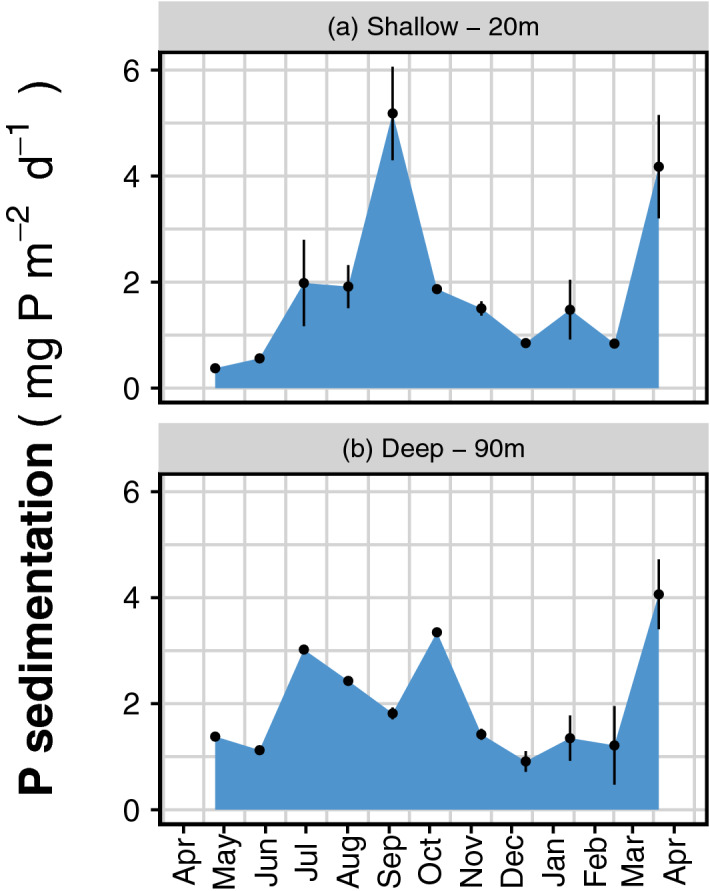
Downward P flux rates determined from total P in settled material caught in automatic sediment traps positioned at 20 m (**a**) and 90 m depth (**b**) at station B from April 2016 to April 2017. Individual collector bottles received material over 31-day periods. Bars indicate ranges from duplicate collector bottles. Total sedimentation was 396 and 375 g dry weight m^−2^ year^−1^ for traps in 20 and 90 m, respectively. Additional data can be found in Supplementary Table S1.

We measured the P fluxes between pools using automatic sedimentation traps collecting settled material over 31-day intervals. The traps estimated the TP fluxes out of the euphotic zone (surface trap at 20 m) and into the oxygen-free deep layer (bottom trap at 90 m). In the surface trap, we found material with more TP (68% of yearly flux) during the 6 months of spring and summer (March–September; Fig. 3). The shapes and integrals of the settling detritus time series were similar for both the surface and deep traps. Thus, only a small amount of the settling material was retained in the first two water compartments.

We determined fluxes across the sediment–water interface from centimetre-scale pore water SRP concentration profiles recorded in April and October 2016 using passive pore water samplers. We area-weighted the flux estimates based on the SRP profiles of stations A–C and derived a lake-wide estimate for the sediment release of SRP into monimolimnetic waters (Table 1). Assuming that the flux calculations represented the sediment release in the specific sub-basin at each station (Table 1), we established that the average monimolimnion-wide flux was 2.15 mg P m^−2^ day^−1^.

The SRP release flux out of the sediment resembled the P sinking flux towards the monimolimnetic waters (bottom trap; 2.01 mg P m^−2^ day^−1^). We expected that most of the material entering the monimolimnetic waters sank to the sediment surface. Thus, the observed similarity between the P sedimentation and SRP release rates suggests that most of the settled P-bearing material was recycled. We assessed the mobile P content of sediment and its compositional changes during diagenesis to confirm the relatively low sediment P retention.

**Figure 4:**
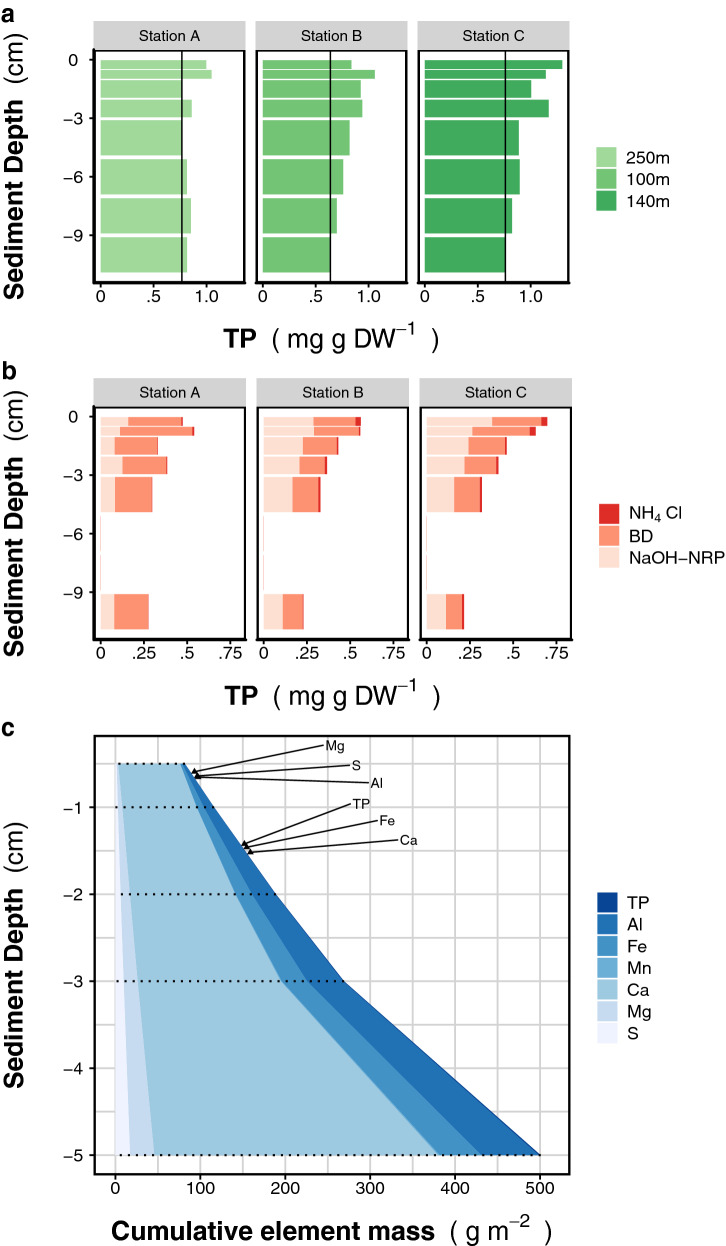
Sediment characteristics of Lake Iseo. (**a**) Total phosphorus (TP) concentration (mg per grams of dry weight) in sediment layers below the sediment–water interface. The black vertical lines indicate a background concentration and exceeding TP is considered to be “mobile P”. Values for sediments between 5 and 9 cm at station B are missing and were interpolated from values above and below. (**b**) Mobile TP fractions differentiated according to a sequential fractionation in redox-sensitive P (BD), free and loosely adsorbed P (NH_4_Cl), organic P and poly-P (NaOH-NRP)^74^. Note scale differences between the panels (**a**) and (**b**). (**c**) Content of selected sediment constituents in surface sediment (Station B). Labeled arrows indicate the specific equilibrium depth up to where the cumulated dry mass contains the same amount of an element as did the settling material collected over one year by the shallow trap. For Mn this value is out of range. Cumulative mass profiles, and additional data can be found in the Supplementary Figure S1 and Table S1.

**Figure 5:**
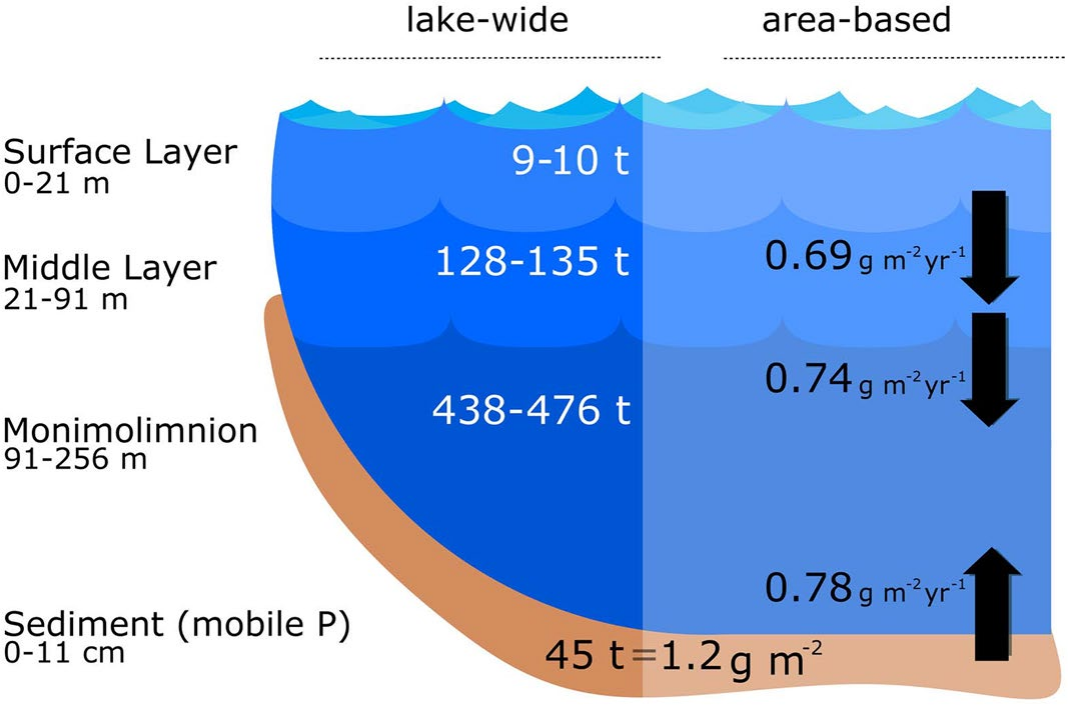
Pools (ranges of 2017) and fluxes (April 2016–April 2017) of total phosphorus (TP) in Lake Iseo. Lake-scale pools are expressed in metric tons (t) of P, and fluxes (right, in g P m^−2^ year^−1^) represent yearly averages. Potentially mobile P (“gradient method”) in the monimolimnetic sediment is expressed both as total and per area. Additional information can be found in Table 1.

We evaluated the sediment records to establish whether they reflected effective P recycling. We identified the size and speciation of the phosphorus-bearing sediment fractions at each station (Fig. 4a,b). Sediment TP decreased with depth to a baseline TP concentration of 0.72 ± 0.07 mg g DW^−1^ (± SD). Any P above this baseline (Fig. 4a; black lines) represented mobile P (“gradient method”; Table 1). We also estimated mobile P by chemically fractionating the sediment and quantifying the P associated with each fraction. The cumulated P bound to the sediment fractions that are generally considered to be mobile (BD, NH_4_Cl, and NaOH-NRP; Fig. 4b) independently quantitates potentially mobile P (“mobile fractions”; Table 1). Most of the mobile P was in the BD and NaOH-NRP fractions and was, therefore, either organic- or Fe-bound P. The P cumulated across all mobile fractions exceeded the mobile P determined from the TP gradient (2.68 g m^−2^ and 1.22 g m^−2^, respectively). Regardless of approach, however, the mobile P estimates were low compared to the TP estimates for the water column. The available mobile TP in the sediment under the monimolimnion was only 7–8% of the water column TP across the lake (45 t vs. 582–613 t; Fig. 5). Therefore, the P was efficiently released from the sediment.

We also used the element-specific contents of collected trap material and subjacent sediment cores from station B to study diagenesis and element recycling. The total P gradients (Fig. 4a) indicated that the diagenesis endpoint was reached in sediments accumulating to ~ 9 cm depth but at 5 cm the major part of mobile P was already lost. The cumulative sediment mass down to 5 cm (2,704 g m^−2^; station B) was 7.2 × greater that the annual sedimentation rate (375 g m^−2^ year^−1^, Supplementary Table S1). If the measured sedimentation flux was similar in past years, the accumulation had occurred over 7.2 year. The same 5 cm layer contains 2.4 g m^−2^ TP which was far less than the amount that had settled during the same accumulation period (5.4 g m^−2^, Table S1). The P had accumulated in the sediment at the rate of 0.33 g P m^−2^ year^−1^ which was equivalent to 45% of the measured input flux. Therefore, the remaining 55% had been recycled and released as SRP.

Similarly, recycling of any sediment constituent may also be elucidated from changes in the elemental composition of the settled detritus during early diagenesis. We compared the 1-year trap material accumulation with the uppermost sediment layers and found that the former contained the same amounts of aluminium, sulphur, and magnesia as the top 6 mm of sediment (Fig. 4c; Table S1). However, relative to the accumulation of these conservative elements, phosphorus and the redox-sensitive metal Fe were depleted in the sediments. To balance equal quantities of the aforementioned elements that settle within 1 year, it was necessary to consider sediment columns of 14 mm (P) and 15 mm (Fe) (Fig. 4c). These again indicated strong release mechanisms for P and other elements involving redox reactions that mineralise organic matter (OM) in the sediments.

We also investigated sediment mineralisation because it maintains steep SRP gradients and high release rates. Areal OM mineralisation was based on the fluxes of reduced-mineralisation metabolites including Mn^2+^, Fe^2+^, S^2−^, CH_4_, and NH_4_^+^^15^. On a mg O_2_ m^−2^ day^−1^ basis, the redox-normalised cumulative flux (F_red_; Table 1) increased from stations C to A to B, similar to the release fluxes that were calculated from the SRP gradients (“P release, October”; Table 1).

Our results show that the sediments in Lake Iseo are transformation, release, and short-term and permanent retention sites in the P cycle. Figure 5 and Table 1 summarise our estimates of the P pools and fluxes between compartments.

## Discussion

In this study, we investigated the influences of deep lake stratification and partitioning into mixed and perennially stagnant compartments on phosphorus (P) distribution and dynamics. We combined the data from an extensive field campaign and quantified the major P pools in Lake Iseo. We also measured the fluxes between its major benthic and pelagic compartments. We then used these data to generalise P controls in meromictic lakes and deduce the potential consequences of shifts in mixing regime caused by warming.

### P dynamics in meromictic Lake Iseo

The measured TP concentrations were consistent across all three stations of Lake Iseo (Fig. 2e/f). Hence, there was relatively homogeneous P distribution in the central part of the basin and we could make robust lake-wide estimates (Fig. 5). The P content in the deep monimolimnion was markedly greater than that of the combined epilimnion and hypolimnion. We addressed several processes contributing to reactive P accumulation in the water column.

The major deep-water P vector is sedimentation. Each year, fourfold more P is continuously removed by sedimentation (733 mg P m^−2^ year^−1^) than that which is retained at steady state in epilimnetic waters during summer (Oct. 2017, 10.18 t P in a 21-m surface layer or 181 mg P m^−2^). The cumulated sedimentation collected between April 2016 and April 2017 (375 g m^−2^ year^−1^; Fig. 3) resembled that of the annual average over the recent decades (265 g m^−2^ year^−1^) reported for the same lake section in a paleolimnological study^41^.

Sinking particles must be labile to benthic processing such as OM mineralisation in order to contribute to bottom-water SRP concentrations. During diagenesis, organic P is depleted. There is relatively less P in the NaOH-extractable fraction of the deeper layers (Fig. 4b). Here, we found that the gross P release fluxes and the areal OM mineralisation rates presented with similar variations across all sample stations. Therefore, much of the settled P is organic and it is recycled by benthic metabolism.

A typical constraint on SRP release to bottom waters is redox-driven, SRP-binding Mn and Fe (oxyhydroxide) formation and dissolution^25^. Temporary oxygen depletion at the sediment surface and in the hypolimnion of productive lakes control the stability of these phases and regulate temporal SRP release. As this process is reversible, however, it does not determine the magnitude of the long-term sedimentary P sink^26,42^. In monimolimnetic sediments, the permanent lack of oxygen and the instability of solid Fe/Mn (oxy)hydroxides deactivate even the temporary SRP storage/release. Thus, the measured release fluxes should not be constrained by redox conditions and should follow a temporal pattern driven by the supply and mineralisation of freshly settled material.

The areal sediment P release flux interpolated from two time points was about as high as the gross sedimentation rate. Thus, the P recycling efficiency, i.e., the ratio of gross release to gross sedimentation, was very high. Anoxic sediments have high P recycling efficiencies (up to 60%)^43^. However, as our sediment surveys in Lake Iseo show, they also retain permanent (non-mobile) P and recycle ~ 55% of the P. Therefore, the release rates (Table 1) were slightly overestimated possibly because of spatiotemporal extrapolation. Our P retention estimates were based on the rates determined for the main basins of Lake Iseo. Nevertheless, retention may vary with magnitude and form of sedimentation and, by extension, distance from tributaries. To confirm that inflow region processes did not bias our estimates, we conducted another sediment survey along a north–south transect (Supplementary Table S4). There were comparatively higher P levels and smaller mobile P fractions in the sediments near the mouths of the two main tributaries. Relatively more poorly mobile P suggested high-density, tributary-imported mineral P (78 t year^−1^)^44^ resistant to recycling. Hence, the TP from sporadically sediment-laden inflow waters rapidly settled in the north basin and only slightly contributed to lake-wide P transformation processes.

**Figure 6:**
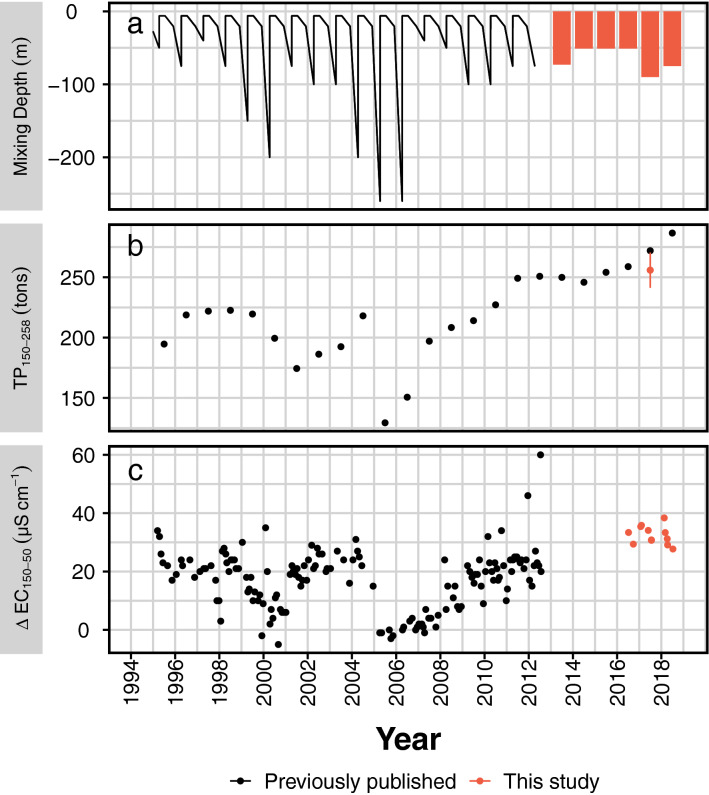
Long term monitoring data from Lake Iseo. (**a**) Mixing depth, shown both as continuous time series until 2012^68^, and as yearly maximum thereafter (M. Pilotti, pers. comm., red bars). (**b**) Total phosphorus in deep waters (150–258 m). Black dots are TP contents (in metric tons) derived from published average TP concentrations^45^. The red bar shows data obtained in this study as range. (**c**) Differences in electrical conductivity (normalized to 25 °C, ΔEC25) between measurements in 150 m and 50 m^47^. Dynamics in ΔEC25 suggest waters from these two depths to experience episodes of decoupling (ΔEC25 increasing), and occasional mixing (ΔEC25 low or zero). Further comparison between EC25 in 50 m and deeper layers can be found in the Supplementary Figure S4. Mixing scenarios are consistent with reports of deep convection in Lake Iseo in 2000, 2005 and 2006^39^, and are accompanied by marked decreases in the deep water TP content.

Our flux measurements showed that rapid turnover occurred in the deep sediments of Lake Iseo and SRP release to the overlying water was expedited. High lake-scale release rates were confirmed by relatively small mobile P pools (44.7 t) in the upper sediment layers. The mobile P rapidly turns over, as without constant renewal it can sustain release (28.7 t year^−1^) for only ~ 1.6 year which is less than in many other holomictic lakes^25^. Long-term studies showed that the TP content in the deep waters of Lake Iseo has increased since the full overturn of 2006 (Fig. 6a/b)^45^. Separation of a monimolimnetic water body combined with high sedimentary P recycling efficiency create intensive water column P accumulation.

### Circulation patterns in a changing climate

As a result of P release from lake sediments, water circulation is an important surface primary production control. Lake circulation patterns are seriously perturbed in warming climates. Longer, warmer summers prolong stagnation by extending water column stability^16, 17^. In turn, stagnation promotes dissolved phosphorus accumulation in bottom waters. However, mixing homogenises the water, a process shown to trigger intense phytoplankton growth pulses in polymictic lakes^46^.

Nevertheless, the aforementioned mechanism does not fully explain the observed nutrient dynamics characteristic of Lake Iseo. Here, a warming climate affects both the timing and extent of mixing^40^. In 2016, the maximum mixing depth was ≤ 60 m^39^ and < 11% of the water column P was circulated. Since the last full overturn of Lake Iseo in 2006, much of the P has been separated from the flushing surface waters and has accumulated primarily in the monimolimnetic waters (instead of the sediments). By comparing the mass of P in the monimolimnetic waters (> 91 m) during holomixis (2005; 255 t)^39^ with that measured for April 2017, we determined an average rate of increase of 18.4 t P year^−1^. This value was lower than the observed rate of release to the monimolimnion (28.7 t P year^−1^; Table 1). Despite the possible over-estimation of the release rate due to spatial and temporal averaging, the difference suggests on the one hand that diagenesis is the primary source of deep-water SRP, and, on the other hand, that this SRP is occasionally lost to the surface waters. Dissolved P may be redistributed to the mixing zone when annual climatic variations shift the depth of the mixing layer (i.e. the monimolimnion surface depth). Long-term data may reveal irregular interaction patterns between deep and surface waters^47^. The occurrence of events wherein hypolimnetic (50 m) and monimolimnetic (150 m) waters have similar electrical conductivities suggests that these layers irregularly exchange solutes including SRP and reduce the P content in the underlying water masses (Fig. 6b,c; Supplementary Fig. S4).

Occasional convection in P-enriched monimolimnetic waters demonstrates that circulation patterns are not exclusively determined by bathymetry^48^. Rather, they are dynamic events that are highly influenced by the local climate^49^. Recurring deep convection that does not extend to the bottom is an extreme result from the general tendency towards increasing water-column stability. This phenomenon has been reported for alpine lakes^35,50,51^, small temperate lakes^52^, and deep lakes in Africa^4^, North America^32^, and Europe^53,54^. Boehrer et al.^22^ showed that incomplete lake overturn can occur in warm-temperate climates and regions where surface waters cool to < 4 °C. Deep lakes slowly accumulate heat in their bottom waters which inevitably leads to deep circulation during cold winters^55^. Thus, deep lake warming promotes a circulation regime that features two characteristic phases: upwards expanding meromixis, followed by its irregular retreat to deeper layers. The latter may induce periodic deep circulation or holomixis.

**Figure 7:**
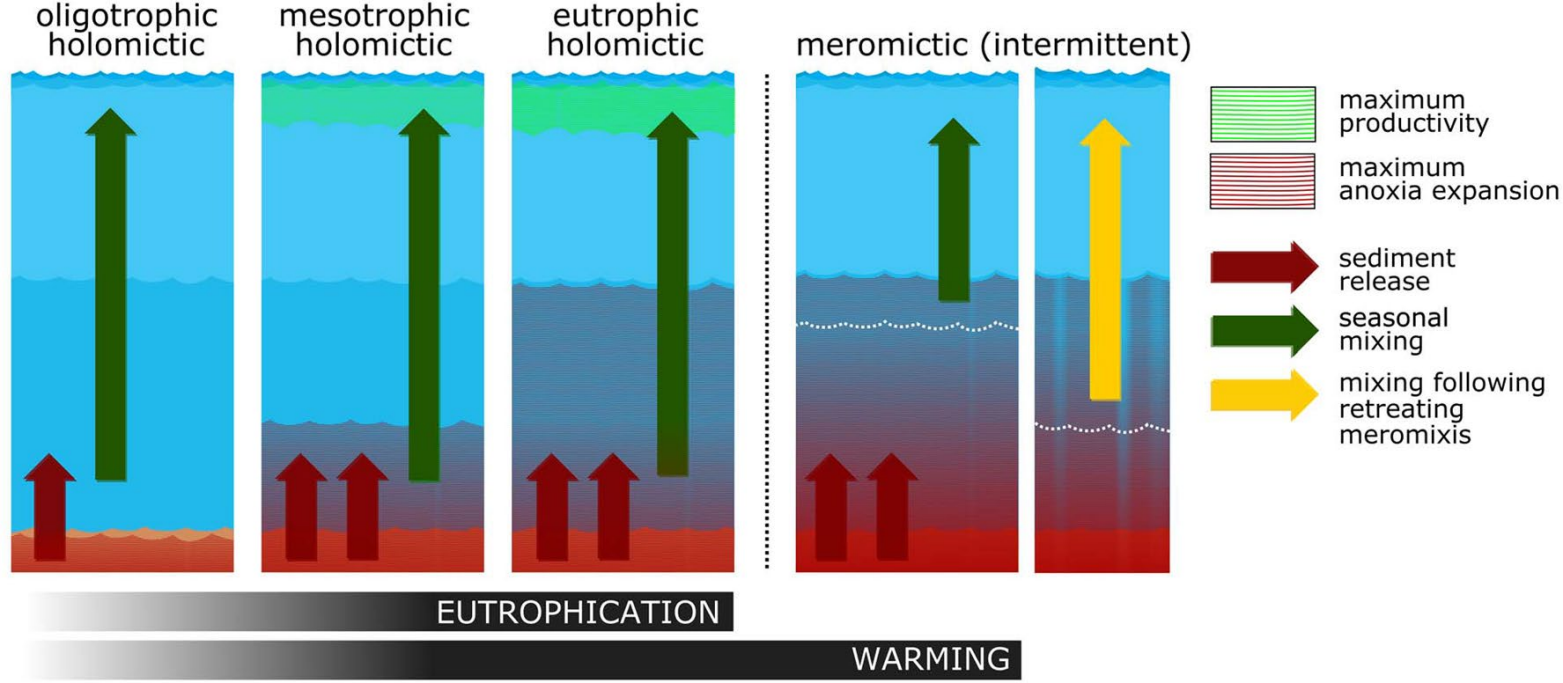
Conceptual illustration of the combined effects of eutrophication and climate forcing on lake trophy. In the holomictic case, stratification first separates epi- and hypolimnion (shades of blue) and transports P from deep waters to the surface upon yearly mixing (green arrow). More P may be transported when larger shares of deep sediments are in anoxic waters. A shift towards warming-induced meromixis could first induce a non-linear response: surface re-oligotrophication together with persistent deep-water anoxia. However, nutrient accumulation in the deep waters bears the potential to trigger episodes of high productivity when meromixis suddenly retreats, allowing surface waters to mix deeper (yellow arrow), as for example during an exceptionally cold winter.

How can such circulation pattern affect lake trophy? Intensive P transfer to deep waters constrains epilimnetic productivity. Consequently, a relatively smaller proportion of P resides in the food web and/or in dead, slowly decomposing organic material. In this way, lake-scale P speciation shifts from particulate to dissolved. Conversely, downwards mixing zone expansion during a single cold winter may introduce pulses of dissolved P originating from several successive years of organic matter decay. Progressing climate change might intermittently perturb nutrient cycling in the deep lakes of all climate zones, including (but not limited to) prolonged episodes of “climate warming-induced oligotrophication”^53^ (Fig. 7).

The observed TP distribution in Lake Iseo is consistent with the aforementioned conceptual model. The negative trend in long-term surface TP data since the last full circulation implies that Lake Iseo is in a state of oligotrophication. However, this trend was not significant^39^. During the same time, however, deep-water TP concentrations have significantly and drastically risen^39^ (Fig. 6), as of 2017 to a water column average of 76 µg L^−1^ (TP_tot_/Vol_tot_, Table 1). This average concentration would be attained across the entire water column after a full circulation. Pulse P introduction to the euphotic zone can trigger mass phytoplankton growth followed by hypoxia and fish kills. A single study surveying this relapse to holomixis in temporarily meromictic lakes reported that the actual ecosystem disturbances were less severe than expected. The termination of 40-year meromixis in Lake Lugano intermittently spiked water column P levels but did not markedly stimulate primary production^28^. The authors proposed that high Fe–P precipitate loads in oxic waters and N limitation after a denitrification surge mitigated the ecological impact of arrested meromixis. Whereas a sudden transformation to holomixis may have only minor short-term effects, re-oligotrophication caused by warming has a severe, long-term impact on a lake ecosystem. A reduction in internal P recycling modulates algal community composition and promotes the proliferation of cyanobacteria benefiting from deep light penetration^29,30,53^. In less productive systems with elevated cyanobacterial densities, the quantity and quantity of food available to fish both decline^56^. At the same time fish habitats shrink due to increasing anoxia and temperature. Thus, climate-induced meromixis in deep lakes has detrimental impacts on fisheries^4^.

### Global assessment

**Figure 8:**
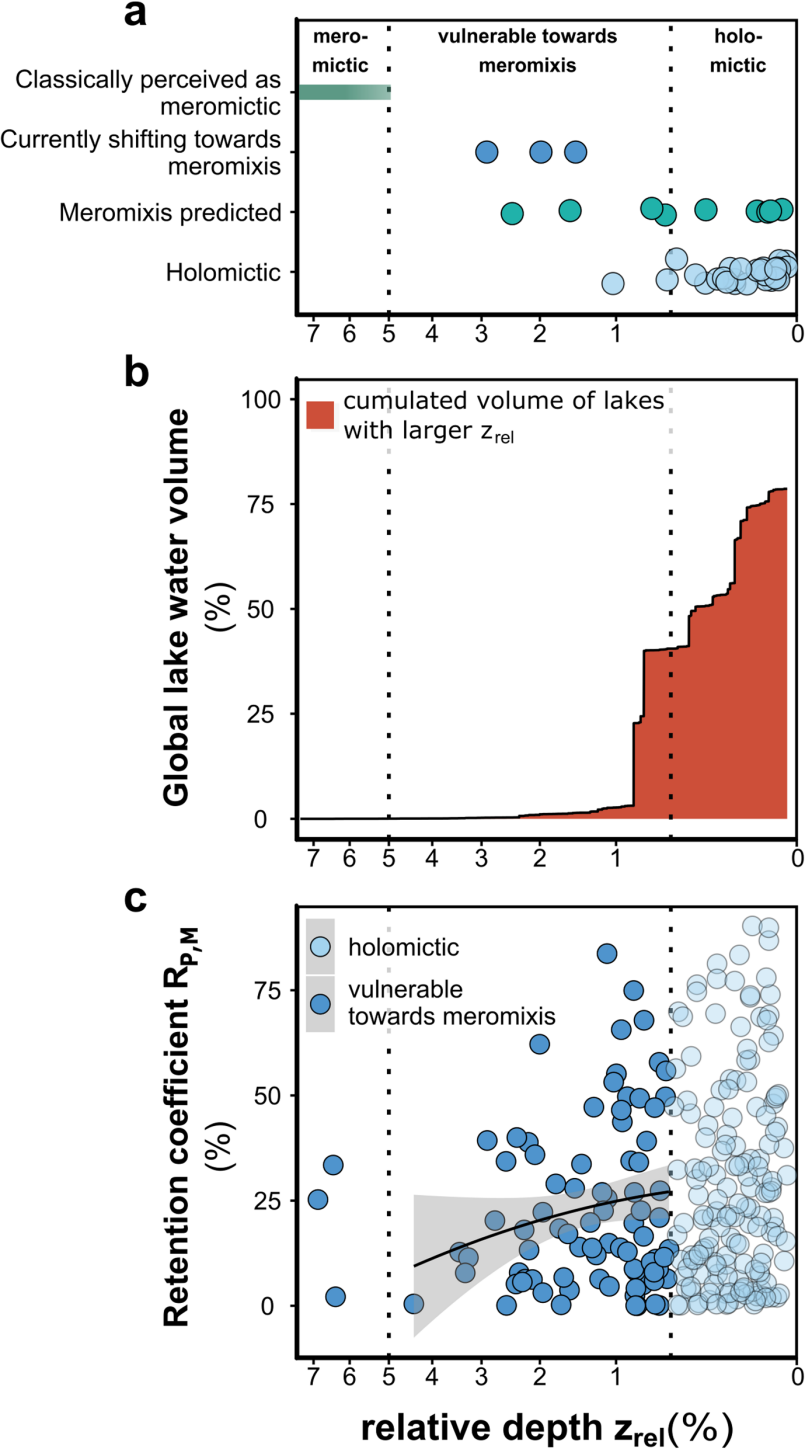
Assessment of the potential for climate-induced meromixis development and the entailing consequences for lake-internal nutrient cycling. The dotted lines illustrate our hypothesis that lakes with a relative depth z_rel_ between 5% and 0.49% have a non-zero chance to be or become meromictic in a warmer world. (**a**) A conservative threshold of 5% is assumed to delineate holo- from meromictic lakes but already today the Lakes Iseo, Bourget and Zurich (z_rel_ 2.90%, 1.98%, 1.49%) feature multi-year deep water stagnation. Rows below show data from Woolway and Merchant^20^ who analyzed lakes (n = 85) for their potential to remain holomictic or shift towards meromixis in a warmer future. (**b**) z_rel_ of 3,475 global lakes included in the GLDBv2^59^. The red area represents the cumulative water volume retained in lakes with a higher z_rel_ relative to the global non-glaciated freshwater resources (excluding the Caspian Sea) (**c**) Lake phosphorus retention^7^ in monimolimnetic waters and sediments when a lake shifts towards meromixis. R_P,M_ was calculated from predicted monimolimnion depths, bathymetry and water residence time of 243 lakes included in both the GLDBv2 and HydroLAKES^78^ databases (Eq. 3b). The black line is a linear model to the data (p < 0.1). Transparent symbols show lakes with a very small chance to turn meromictic (z_rel_ < 0.49%).

Meromixis was formerly considered a stationary limnological characteristic peculiar to lakes with specific features^38,57^. In contrast, recent studies demonstrated that many lakes can change their circulation patterns under a warming climate and present with extreme forms of intermittent meromixis^33^. The perturbation in P cycling observed in Lake Iseo could also occur in lakes of similar morphometry. Salinity distribution must be measured in order to assess the risk of meromixis in a lake. However, Walker and Likens proposed relative lake depth (z_rel_) as a useful proxy and confirmed that lakes with a z_rel_ > 5% are very likely to be meromictic^38^. Nevertheless, the relative depth of Lake Iseo is only 2.90%. Even stronger deviations from this approximation include the climate-induced stabilization with multi-year stagnation, reported for example in Lake Zurich (z_rel_ = 1.49%) and Lac Bourget (z_rel_ = 1.98%)^35,50,54,58^. In the future, the most frequently occurring change in lake mixing might be the transition from holomixis to meromixis. By 2080, this conversion process might also affect lakes with z_rel_ as low as 0.2%^20^. Under the current warming climate trend, the threshold for meromixis development could be weakening (Fig. 8a).

To assess the risk of trophic modulation caused by climate-induced meromixis in global lakes, we used data from the Global Lake Database GLDBv2^59^. It contains morphometric parameters for ~ 13,000 lakes. When only lakes > 1 km^2^ (n = 3,475) are considered, it comprises ~ 50% of the cumulative global lake area^60^ and 80% of the global lake volume excluding the Caspian Sea^61^. Based on these data, the morphometric precondition of Lake Iseo for meromixis development (z_rel_ > 2.90%) is surpassed by only ~0.3 vol% of all global lakes. However, the results of a modelling study suggest that a feasible future threshold could be lower and we propose an alternative threshold based on the lakes for which holomixis is predicted to persist through to 2080^20^ (n = 65 of 80 modelled lakes) and for which there is sufficient morphometric data (contained in GLDBv2, n = 29/65). When the upper bound of z_rel_ (95th percentile; z_rel_ = 0.49%) was taken as the new lower threshold for vulnerability to meromixis, GLDBv2 disclosed that lakes comprising 40% of the global lake water might eventually develop perennial anoxic bottom layers (Fig. 8b).

Most of the global non-glaciated fresh water is stored in a few very large lakes including those wherein deep basin water stagnation affects only a small proportion of the total lake volume. This fraction might, in fact, be many times smaller than that of water bodies such as Lake Iseo that have wide basins. Thus, variability in the affected volume must be considered when estimating the effects of meromixis on lake nutrient balances. To this end, we calculated the fraction of external P loading retained in a body of water (retention coefficient, R_P_)^37,62^ and performed spatial normalisation. The latter considers that the monimolimnion receives only a small amount of the total P loading. This relative quantity is equivalent to the monimolimnions’ fraction of the total surface area (A_M_/A_tot_) which can be calculated based on an empirical formula for monimolimnion surface depth^63^. Monimolimnion P retention calculations (R_P,M_ = R_P_∙A_M_/A_tot_) for 243 lakes (Fig. 8c) revealed that the onset of meromixis in susceptible lakes may trigger the retention of large fractions of P loading (0–83%; median, 17%) outside of the annually mixed layer. Due to the high P recycling efficiency of anoxic sediments, large parts of this P may be released as SRP to irregularly circulated waters^43^. A non-significant (*P* < 0.1) trend in R_P,M_ suggested that when lakes with relatively small z_rel_ are affected, deep-layer P accumulation is intensified, and so are the effects of P redistribution to the euphotic zone when meromixis retreats.

### Implications for lake managers

Under a warming climate, numerous lakes may develop stagnant bottom waters^20^. Wind counteracts water column stabilization^64^ but an offsetting trend in their forcing is uncertain^17^. An objective of this study was to underscore that climate change constrains mixing and modifies nutrient cycling in lakes already under intense pressure from urbanisation and eutrophication^65^.

Warming-induced meromixis presents lake managers with new challenges. Less internal P loading will lead to lower trophy and augment recreational use. In contrast, progressive deep-water anoxia invisibly threatens aquatic organisms. Lake managers may have to weigh contradicting outcomes such as reduced oxygen deficiency vs. increased nutrient loading. Moreover, lakeshore communities might not be motivated to lower local anthropogenic pressure.

Deep convection is a fundamental and ubiquitous driver of ecosystem-scale metabolism. The present study of short-term P dynamics showed that a shift towards meromixis promotes P storage in the form of deep-water SRP. It also disclosed that these shifts may occur in lakes with relative depths < 5%. These findings were consistent with the previously reported observation that lakes and reservoirs keep comparatively more P in their water columns if their relative depth is larger^37^. The results of our study align with those published in an earlier case report^53^. Here, we provided a detailed biogeochemical mechanism explaining the observed decreases in phytoplankton biomass in many deep lakes worldwide^8,66^. To the best of our knowledge, this study is also the first to address the ecological consequences of infrequent but inevitable excursions of circulation depths in deep lakes. In future research, the substitution of lake-specific salinity and density data for z_rel_ as a mixing proxy could accurately predict mixing pattern changes. Clarification of these changes is necessary to be able to identify the drivers, prevalence, and consequences of global-scale warming-induced lake meromixis.

## Methods

### Study site

Lake Iseo (A = 61 km^2^; z_max_ = 256 m) is located in the pre-alpine region of Italy at the southern end of Valle Camonica, a glacial valley 1,440 km^2^ in area. An early limnological study classified Lake Iseo as warm monomictic and oligotrophic^67^. Oxygen was available through the entire water column and the phosphorus concentration was only a few µg L^−1^. Subsequent limnological studies between 1967 and 1992 reported that the water quality of Lake Iseo had deteriorated as a result of eutrophication^44^. A few other studies showed that winter circulation has been incomplete in Lake Iseo since the 1980s^39,68^. Only two complete circulations have been observed in Lake Iseo over the past 30 year. This decreasing trend in deep mixing is explained by the combined effects of relatively milder winters and chemical stratification^69^.

### Field sampling

Highly resolved temperature, electrical conductivity (EC at 25 °C) and oxygen profiles were measured using profilers (RINKO, JFE Advantech Co. Ltd., Nishinomiya, Japan) at stations A (255 m), B (100 m), and C (150 m) (Fig. 1a). The lake bed measurements at these stations were taken to be representative of the sediment layers at three different depth ranges and their corresponding areas (Table 1). Water was sampled in April/October 2017 at 12–20 depths in stations A–C. Subsamples were immediately prepared and passed through a 0.45-µm filter (cellulose acetate; Th. Geyer, Renningen, Germany), preserved according to standard procedures^70^, and subjected to analyses of soluble reactive phosphorus (SRP) and other solutes.

### Chemical analysis

The solutes in lake and pore water, extracts, and digests were analysed by standard methods within a few days after sampling^70^. SRP and ammonium (NH_4_^+^) were determined by segmented flow analysis (Skalar Sanplus, Skalar Analytical B.V., De Breda, The Netherlands). Dissolved metal (Fe, Mn, Mg, Ca) concentrations were determined by inductively coupled plasma optical emission spectrometry (ICP-OES; iCAP 7000 series; Thermo Fisher Scientific, Waltham, MA, USA). Dissolved nitrate and sulphate were quantified by ion chromatography (Shimadzu Corporation, Nakagyo-ku, Japan).

Unfiltered samples consecrated for total P (TP) determination were stored at 4 °C. TP in water samples was measured as SRP after K_2_S_2_O_8_ digestions (5%) at 120 °C. TP content of solid material was determined as SRP after digestion of 5–10 mg dry sediment in a solution of 2 mL 5 M H_2_SO_4_, 2 mL 30% H_2_O_2_, and 20 mL distilled water at 150 °C for 8 h.

### Sedimentation and sediment samples

Vertical downward fluxes of particulate material (sedimentation) were quantified using two automatic cylindrical sediment traps (Hydro-Bios Ltd., Kiel, Germany). Each of these was fitted with 24 collecting bottles and two cylinders (154 cm^2^) for duplicate sampling (Fig. S6). Bottle contents were preserved with pre-added formalin to prevent decomposition of the settled material. One multi-trap was positioned below the epilimnion (20 m) whilst the other was set near the hypolimnion-monimolimnion interface at 90 m. The exposure period started on 8 April 2016 and ended on 5 April 2017 and the interval between samplings was 31 days. The contents of the recovered bottles were concentrated, freeze-dried, weighed, and subjected to total P and elemental (C, N, and S) analyses (Vario EL; Elementar Analysensysteme GmbH, Hanau, Germany). The dried bottle contents were digested with aqua regia in a microwave oven and their total element (Al, Ca, Mg, Fe and Mn) levels were determined by ICP-OES. The sedimentation flux rates (mg m^−2^ day^−1^) of these elements were calculated by multiplying the areal dry mass flux rate by the elemental content in the settled material at each time interval.

The chemical gradients of the dissolved substances above and below the sediment–water interface were determined with 60-cm long dialysis samplers^71^ positioned half-way in the sediment at all stations over 14-day periods. The dialysis samplers enabled concentration profile measurements at 1-cm increments. The samplers were removed from the sediment and the equilibrated water in the chambers was retrieved by piercing their 0.2-µm membranes (HT-Tuffryn 200; Pall Gelman Laboratory, Port Washington, NY, USA) with pipettes.

Undisturbed sediment cores were obtained with a modified Kajak sampler (UWITEC; Mondsee, Austria). The mobile P pool in the sediment was determined by the “gradient method” using duplicate cores at all stations^70^. For this purpose, sediment cores were sliced into layers (0–0.5 cm, 0.5–1.0 cm, 1–2 cm, 2–3 cm, 3–5 cm, 5–7 cm, 7–9 cm, and 9–11 cm). The mobile P pool in the sediment was calculated from the difference between the TP in each layer and the background TP (i.e., the lowest relative P content in sediments down to 11 cm)^70^. It was assumed that sampling down to 11 cm was enough to contain material passing the early diagenesis endpoint. The differences were cumulated according to the dry mass in each layer and yielded the mobile P mass per unit area. The aforementioned sediment layers (excluding 5–9 cm) were fractionated according to a sequential extraction scheme^72,73^. Six operationally defined P fractions were separated as follows: (1) loosely adsorbed P and P in pore water (NH_4_Cl-P); (2) reductant-soluble P bound mainly to Fe-hydroxides (BD-P); (3) P bound to Fe-oxides or Al-oxides (NaOH-SRP); (4) organic P and poly-P (NaOH-NRP); (5) P bound to carbonates and apatite (HCl-P); and (6) refractory P determined after digestion of the remaining sediment (Res-P). The sum of the NH_4_Cl-P, BD-P, and NaOH-NRP fractions represented potentially “mobile” P that contribute to P release^74^.

Methane (CH_4_) levels in the sediments were determined in October 2016 by slicing three cores per station in 12 increments of either 2 cm (0–20 cm) or 5 cm (20–30 cm). Triplicate syringes were used to transfer 2.9 mL sediment from each layer to 20-mL vials containing 0.03 mg ZnCl_2_ to arrest microbial activity. The vials were sealed with PTFE-covered silicon stoppers and stored until autosampler-assisted gas chromatography-flame ionisation detection (GC-FID; Shimadzu Corp., Kyoto, Japan) determination of the methane concentration in the headspace.

### Data analysis and statistics

The concentrations between sampled depths were interpolated. The mass of total P in each 1-m water layer was calculated by multiplying the concentration at the centre of each layer by the layer volume obtained from the hypsographic curve (Figure S1a). The values for all single layers were summed to derive the overall P mass in the surface (0–21 m), middle (21–91 m), and monimolimnion (91–256 m) layers. For long-term time series, the average P concentrations in the 150–258-m layer^45^ were multiplied by the volume of this stratum. The phosphorus release rates and the flux rates of the other solutes and methane were calculated from the concentration profiles across the sediment–water interface according to Fick’s first law^75^ using published diffusion coefficients^76^. With respect to individual redox stoichiometry, the cumulated release rates of ammonium, sulphate, Fe^2+^, Mn^2+^, and CH_4_ corresponded to anaerobic benthic mineralisation as they express the flux of the reduced mineralisation end products (F_red_) in oxygen equivalents (Tables S2 and S3)^15^. Lake-wide estimates for sediment pools and fluxes were derived by weighted averages from measured values as detailed in the Supplementary Informations.

Graphs were plotted and statistical analyses were performed using R v. 3.4.3 (R Core Team, Vienna, Austria)^77^ and the *raster*, *vegan*, and *ggplot2* packages.

To simulate the responses of lake P dynamics to meromixis, the range of monimolimnetic waters and the magnitude of P sequestration were estimated. A dataset of morphometric (maximum depth, D_max_, average depth, D_av_, and surface area, A) and hydrological characteristics (water residence time, τ_w_) for global lakes was compiled. Data were acquired from the open-access datasets GLDBv2 and HydroLAKES^59,78^. Relative lake depth (a "back-of-the-envelope" proxy for susceptibility to meromixis) was calculated according to the method of Håkanson as follows (D_max_ in m, A in km^2^ return z_rel_ in %):^79^

1 $$z_{rel} = \frac{{D_{max} \sqrt \pi }}{20\sqrt A }$$

A subset of 243 lakes was included in both datasets (the name and location were used as pairing criteria). This subset was applied to calculate the P retention (*R*_P_) according to the mass balance concept of Vollenweider^7^. In such mass balance models, all TP that enters and remains in the system is considered as P retention, expressed as unitless factor, *R*_*P*_ (–). P retention can be calculated without knowledge of a lake’s P load (i.e., its inflow concentration *TP*_*in*_) assuming the latter to be constant:

2 $$R_{P} = \frac{{TP_{in} - TP_{out} }}{{TP_{in} }} = 1 - \frac{1}{{1 + \sigma \tau_{w} }}$$

where $\sigma$ is the first-order rate of TP loss via net lake sedimentation. It was approximated as a constant value of 0.45 year^−1^^62^. Much of the phosphorus load to a lake is retained through sedimentation^37^, and settling into the monimolimnion separates P from the mixed layer. Therefore, the same mass balance may be used to derive the retention *R*_*P*_ of the mixed layer and of the stagnant layer, respectively. Assuming homogeneous sedimentation throughout the lake, the fraction of the phosphorus retention realised in mixed water layer sediments was calculated as follows:

3a $$R_{P,m} = R_{P} \frac{{A_{m} }}{A}$$

where $A_{m}$ is the difference between the total and monimolimnion surface areas. The remaining fraction of the phosphorus retention factor $R_{P,M}$ is realized in the monimolimnion and the P was therefore retained in its waters and sediments. It was calculated as follows:

3b $$R_{P,M} = R_{P} \frac{{A_{M} }}{A}$$

The area of the monimolimnion surface A_M_ was computed with a bathymetry-based model for meromictic lake stratification^63^. (See the Supplementary Information and Eq. S1–S3). $R_{P,M}$ in Fig. 8c illustrates the relative extent of phosphorus retention in the monimolimnetic compartment of a formerly holomictic lake that has become meromictic.

## Supplementary information

Supplementary Information 1.

## Data Availability

The authors declare that data supporting the findings of this study are available from the authors upon request. Datasets GLDBv2^59^ and HydroLAKES^78^ were obtained from their online repositories.
