# Supplementary material for: Intermittent meromixis controls the trophic state of warming deep lakes
Source: Sci Rep. 2020 Jul 31;10:12928. doi: 10.1038/s41598-020-69721-5 (PMC7395808; doi:10.1038/s41598-020-69721-5)
Supplement: Supplementary file 1 — Supplementary Information 1. [file 41598_2020_69721_MOESM1_ESM.pdf]

Supplementary Information

Intermittent meromixis controls the trophic state of warming deep lakes

Maximilian P. Lau[1,3]*, Giulia Valerio[2], Marco Pilotti[2], Michael Hupfer[1]

[1] Leibniz Institute of Freshwater Ecology and Inland Fisheries (IGB), Department of Chemical Analytics and Biogeochemistry, Müggelseedamm 301, D-12587 Berlin, Germany
[2] Università degli Studi di Brescia, Dipartimento DICATAM, Via Branze 43, I-25123 Brescia, Italy
[3] Université du Québèc à Montréal (UQAM), Département des sciences biologiques, 141, avenue du Président-Kennedy, Montréal, Québèc (H2X 1Y4), Canada

* Corresponding author

Content

1. Basin bathymetry
2. Composition of sediments in trap bottles and cores
3. Mineralization rate estimate
4. Sediment characteristics across Lake Iseo
5. Deep mixing effect on EC25
6. Global meromixis prevalence and impact computation
7. Methods used for P pool and flux determination

1 Basin bathymetry

To account for spatial heterogeneity we collected samples at three locations (Station A-C). In general, we found water column parameters to be relatively similar across stations (Figure 2). In contrast, sediment parameters differed more strongly (for example in dry mass and P content), likely because of different sediment compaction (the stations have different depths) and settling flux composition (the stations have different distance from the main inlet). Consequently, the basin was partitioned so that sub-basins can adequately reflect these differences. In brief, the results from Station A represent the deep sediments, because this basin (Central/North) includes all sediments below 175m, while Stations B and C represent the sediments that form within the shallow channel created by the island (East Channel), and the sediments that form in maximum distance from the main inlet (South), respectively. Therefore, we expect the most accurate lake-wide estimates when we treat the three stations not as replicates but as representatives of these specific sub-basins. The bathymetric curves of the sub-basins are shown in Figure S1 and they accumulate to 100% of the area of Lake Iseo (Figure S1a). Lake-scale estimates of sediment characteristics (e.g. fluxes or pools) were derived by multiplication of values obtained in each station with the sub-basin's respective fraction of the total monimolimnion surface area (i.e. area below 90m, Table 1), followed by addition of these three local values.

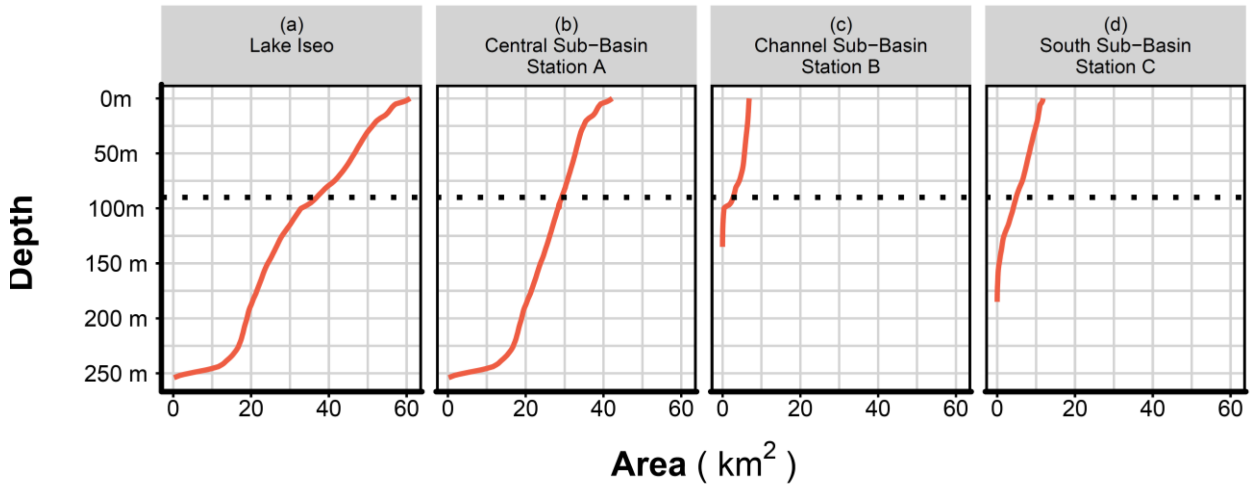

Figure S1 Hypsographic curves of Lake Iseo shown for the whole lake (a) and for all sub-basins (b-d). Area below the dotted line (91 m) is considered as the monimolimnion area (2017), in each basin represented by one of the measurement stations.

2 Composition of sediments in trap bottles and cores

Element-specific content of collected material from traps and the sediment that formed below (from sediment cores, all at station B) are a convenient system to study sediment diagenesis and element recycling (see discussion section).

Table S1 Selected element contents of sediment material taken in sediment cores or collected with traps from the water column (Station B, 2016-2017).

Material	Unit	Total Dry Mass	TP	Al	Fe	Mn	Ca	Mg	S
Trap 20m (accumulated over full year) [a]	$g\ m^{-2}\ yr^{-1}$	395.9	0.7	6.7	13	0.1	110.2	2.5	1.4
Trap 90m (accumulated over full year) [a]	$g\ m^{-2}\ yr^{-1}$	374.6	0.7	7.3	5.5	3.2	26.3	3	1.5
Sediment 0 - 0.5 cm	$g\ m^{-2}$	329.8	0.3	3	2.4	0.2	72.9	1.8	1.3
Sediment 0.5 - 1 cm	$g\ m^{-2}$	222.5	0.2	8.6	5.9	0.2	14.5	3.4	1.5
Sediment 1 - 2 cm	$g\ m^{-2}$	459.4	0.4	15.7	10.8	0.4	36.4	5.8	3.2
Sediment 2 - 3 cm	$g\ m^{-2}$	469.6	0.4	14.8	10.8	0.4	44.6	5.6	3.6
Sediment 3 - 5 cm	$g\ m^{-2}$	1 223.2	1.0	25.4	19.0	0.8	165.7	12	7.6

[a] Sum of monthly averages from duplicate sample bottles

3 Mineralization rate estimate

Porewater concentration gradients and the according fluxes were determined as described in the methods section. Table S2 shows the individual fluxes of the reduced mineralization end products. Mineralization of carbon in organic matter (C_{org}) may proceed according to

$$C_{org} + O_2 \rightarrow CO_2$$

, assuming an average carbon oxidation state of 0. To infer the amount of organic matter mineralized in the absence of oxygen, fluxes of anaerobic metabolites may be normalized according to their oxidation stochiometry (Table S3).

Table S2 Fluxes across the sediment-water interface on stations A, B and C (spring of 2016)

**mmol m^{-2} d^{-1}**			
**Station**	A	B	C
**NH_4^+**	-0.72	-1.28	-0.76
**Fe(II)**	0.00	-0.01	0.00
**Mn(II)**	-0.01	-0.02	-0.01
**CH_4**	-3.05	-3.41	-2.33
**H_2S**	n.d.	n.d.	n.d.

n.d. = not determined

Table S3 Oxidation stoichiometry to calculate sediment oxygen consumption

**X**	**Molecular mass (g/mol)**	**Stoichiometry (mol O_2 eq. / mol X)**
**NH_4^+**	18	2
**Fe(II)**	55.8	0.25
**Mn(II)**	54.9	0.5
**CH_4**	16	2
**O_2**	32	1

4 Sediment characteristics across Lake Iseo

Analysis of sediment composition along a North-to-South transect in Lake Iseo. Main tributaries are in the North basin.

Table S4 Areal abundance of selected sediment components along a N-S transect in Lake Iseo.

**Station** (in flow direction)	**P content** mg P m^{-2}		**C content** g C m^{-2}		**mobile TP fractions** [% tot.][c]	
**Sediment horizon [cm]**	**0-1**	**1-5**	**0-1**	**1-5**	**0-1**	**1-5**
**B4** [b]	2375	17327	119	824	48%	28%
**B5** [b]	2987	14046	124	710	43%	25%
**B6** [b]	1014	4697	61	331	61%	50%
**A** [a]	1493	8634	120	861	54%	43%
**B** [a]	512	2873	58	316	65%	53%
**C** [a]	552	3048	52	285	72%	50%
**B7** [b]	887	2588	75	327	71%	59%

[a] regular station with detailed parameters for sediment speciation [b] additional stations with data for two sediment layers [c] sum of redox-sensitive P (BD), free and loosely adsorbed P (NH_4Cl), and organic P and poly-P (NRP) as percentage of total P in all sediment fractions

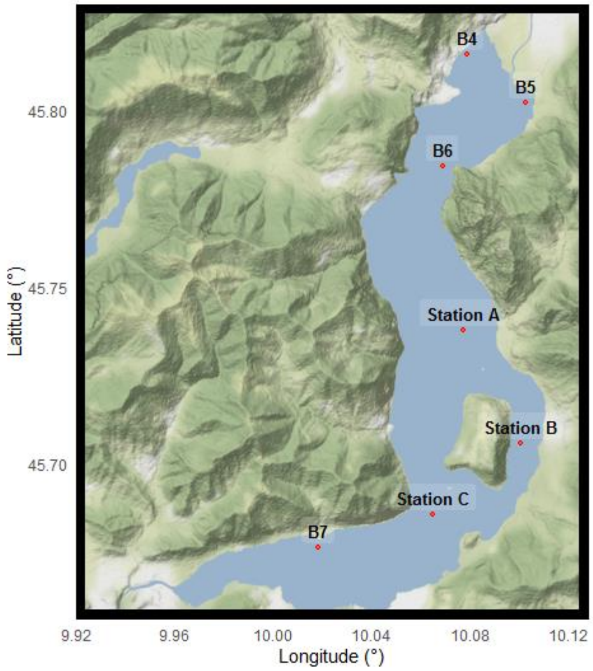

Figure S2 Additional sediment sample locations. Map was prepared by R software (Version 3.4.3)[1] using the package ggmap and map tiles from *Stamen Design (CC BY 3.0).*

Figure S3 shows sediment dry mass profiles. Cumulative dry mass may vary with sediment composition and depth. Cores from larger depths have a tendency to be more compacted with a higher dry mass per volume.

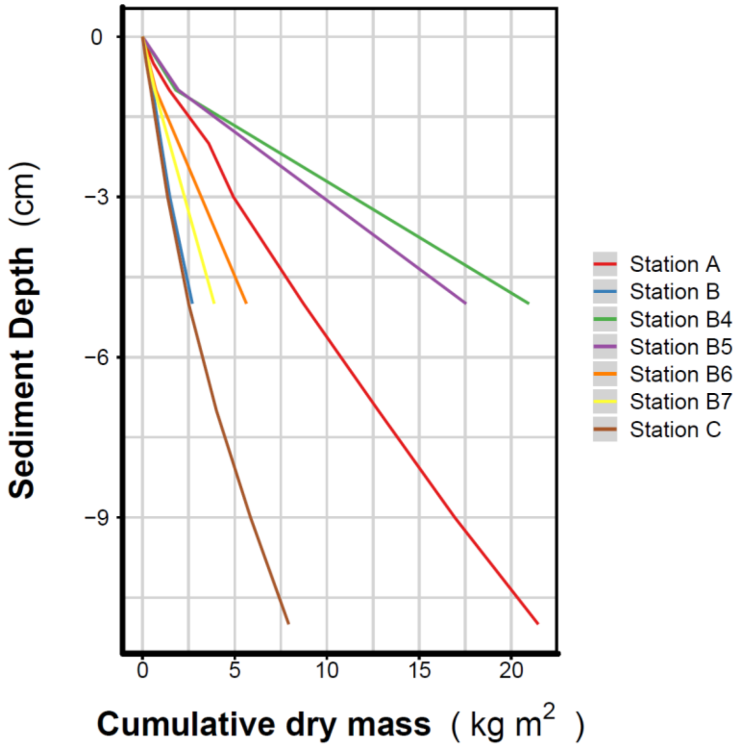

Figure S3 Cumulative dry mass profiles of sediment cores across all stations.

5 Deep mixing effects on EC25

We show in Figure 6 that deep-water SRP content drop when surface and deep layers mix. We infer the timepoint and magnitude of solute exchange from published conductivity and mixing depths time series.[2] Episodes, during which waters from the middle layer (50m) and the monimolimnion (e.g., 150m) mix show similar electrical conductivity, suggesting these waters are in a state of connection and solute exchange. Using the data from the same source, we expanded this analysis to other water layers between the depth of 100 and 245m. Figure S4 illustrates how conductivity (EC25) of waters are more likely to be similar (i.e., close to the 1:1 line) during and shortly after mixing events that reached down to the given depths.

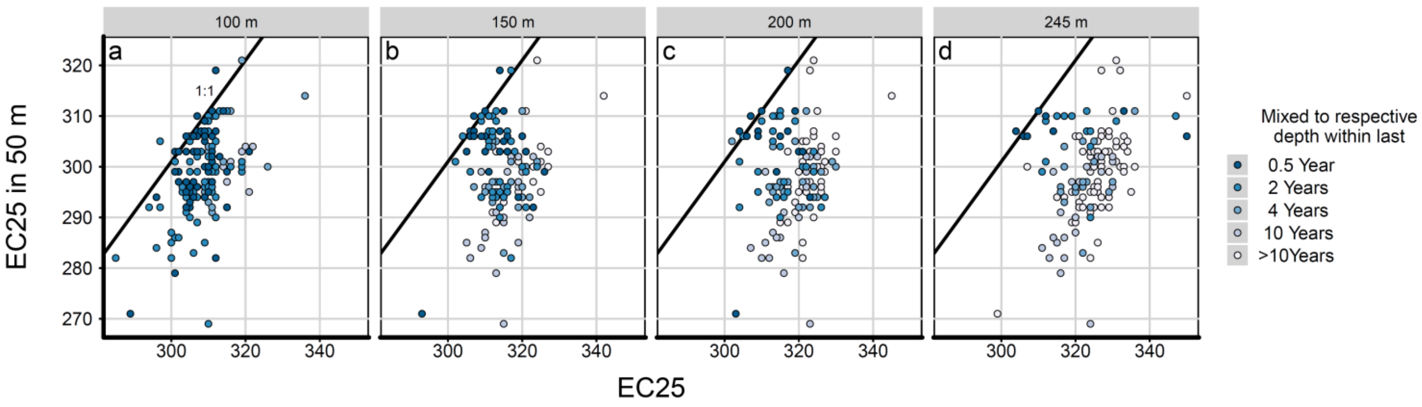

Figure S4 Comparison of electrical conductivity (normalized to 25°C, EC25) in four different water depths (a-d) with EC25 closer to the surface (50m). Data was obtained from published EC25 time series.[2] Black lines show identical values. Symbols are EC25 measurements between 1995 and 2012. Colors indicate the time that passed since the last mixing event that reached at least to the respective depths. Daily modelled mixing depth was available from 1977 to 2012.[3]

6 Global meromixis prevalence and impact computation

Data for global lake bathymetry (maximum depth, medium depth, location, surface area) was obtained from the GLDBv2 dataset. We excluded lakes for which the mean depth was either not included or estimated by a simple approximation (volumetric factor of 1.500). The depth of the upper surface of the monimolimnetic water layer D_{mml} was derived based on an empirical model formulated by Bryhn[4]. He suggested that this D_{mml} may be used as a reference of the interface between monimolimnion and mixolimnion (i.e., the water layers that circulate in regular intervals) that can be expected to appear in meromictic lakes, irrespective of the reason for meromixis.

$$D_{mml} = 6.70 \cdot \frac{{D_{max}}^{1.23}}{21.4 + \sqrt{A}} \qquad \text{(S1)}$$

Using this depth, for each lake we calculated the surface area of the monimolimnion from the bathymetric model of Håkanson[5]. To this end, the author uses the volumetric factor for each lake, a geometric relationship between the lake volume and surface area as follows[5]:

$$V_d = 3 \cdot \frac{D_{av}}{D_{max}} \qquad \text{(S2)}$$

$$\frac{A_M}{A} = \left(\frac{(D_{max} - D_{mml})}{(D_{max} + D_{mml} \cdot \exp(3 - {V_d}^{1.5})}\right)^{\frac{0.5}{V_d}} \qquad \text{(S3)}$$

with V_d as the volumetric factor, D_{av} the average depth, D_{max} the maximum depth, and $\frac{A_M}{A}$ (0-1, no unit) as the areal fraction of monimolimnion surface area relative to the total lake surface. The absolute monimolimnion surface area can be derived by simple multiplication.

Accordingly, if sedimentation is assumed to be spatially homogeneous across the surface waters of a lake, $\frac{A_M}{A}$ equals the share of sediment material diverted towards non-mixing monimolimnetic waters (see discussion section).

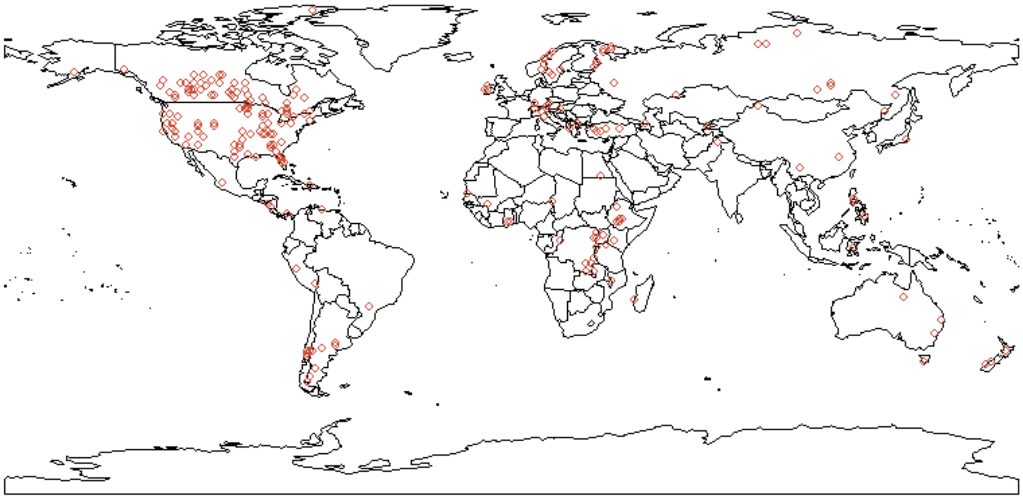

Figure S5 Location of lakes included GLDBv2 and hydroLAKES datasets (n=243). Map was prepared by *R* software (Version 3.4.3)[76] using the package *rworldmap*.

7 Methods used for P pool and flux determination

Sampling strategies were carefully designed to produce estimates for phosphorus pools in sediment and various water layers of Lake Iseo, as well as the fluxes between these pools. Figure S6 illustrates the methodological portfolio with which the presented results were obtained.

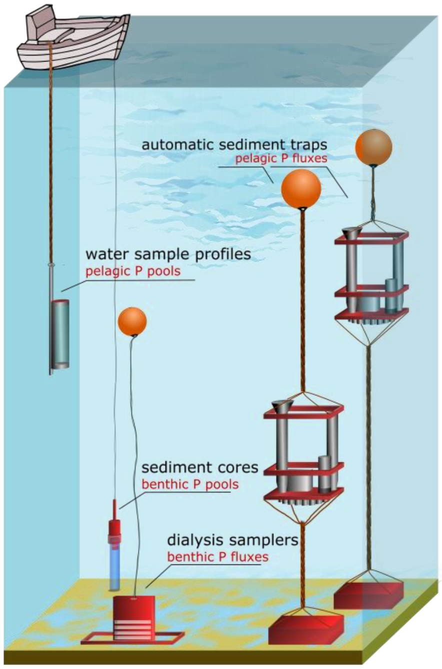

Figure S6 Sampling strategies for P pools and fluxes in Lake Iseo.

References

1. R Core Team. R: A language and environment for statistical computing. (2013).

2. Pilotti, M., Valerio, G. & Leoni, B. Data set for hydrodynamic lake model calibration: A deep prealpine case. *Water Resour. Res.* **49**, 7159–7163 (2013).

3. Pilotti, M., Simoncelli, S. & Valerio, G. A simple approach to the evaluation of the actual water renewal time of natural stratified lakes. *Water Resour. Res.* **50**, 2830–2849 (2014).

4. Bryhn, A. C. A morphometrically based method for predicting water layer boundaries in meromictic lakes. *Hydrobiologia* **636**, 413–419 (2009).

5. Håkanson, L. The importance of lake morphometry for the structure and function of lakes. *Int. Rev. Hydrobiol.* **90**, 433–461 (2005).

(All references are also provided in the main document.)
